# Gender Differences in Motivation and Barriers for The Practice of Physical Exercise in Adolescence

**DOI:** 10.3390/ijerph17010168

**Published:** 2019-12-25

**Authors:** Iago Portela-Pino, Antonio López-Castedo, María José Martínez-Patiño, Teresa Valverde-Esteve, José Domínguez-Alonso

**Affiliations:** 1 Department of Physical Education, University of Vigo, 36310 Vigo, Spain; iagoportt92@gmail.com (I.P.-P.); mjpatino@uvigo.es (M.J.M.-P.); 2 Department of Analysis and Psicoeducative Intervention, University of Vigo, 36310 Vigo, Spain; alopez@uvigo.es (A.L.-C.); jdalonso@uvigo.es (J.D.-A.); 3 Department of Didactics of Music, Visual and Body Expression, University of Valencia, 46022 Valencia, Spain

**Keywords:** physical exercise, motivation, barriers, adolescence, correlation analysis

## Abstract

A total of 852 adolescents between 12 and 17 years of age were evaluated (M = 14.86, SD = 1.67), randomly selected among a population of secondary school and Baccalaureate students. We applied an “ad hoc” questionnaire on sociodemographic data and the Self-Report of Reasons for the Practice of Physical Exercise (AMPEF) and Self-Report of Barriers to the Practice of Physical Exercise (ABPEF) questionnaires. The results obtained show a prevalence of competition, social recognition, and challenge as motivational factors, and fatigue or laziness as barriers to physical exercise among adolescents. With respect to gender, boys show a greater presence of motivations towards doing physical exercise, while girls show more barriers. In conclusion, the motivational factors and barriers interact and support each other for a better predisposition towards physical exercise.

## 1. Introduction

The practice of physical exercise has become an issue of increasing social importance in recent decades because, among other reasons, physical inactivity and sedentary lifestyles have contributed to the growing prevalence of pathologies such as obesity, diabetes mellitus, cardiovascular diseases, hypertension, and stroke, which have become the scourge of modern societies [1]. The rate of these diseases continues to rise, which has led to increased morbidity and mortality and therefore the urgent need to implement effective physical activity programmes to reduce sedentary behaviour among the population [2].

Adolescence is a critical period in the formation of the individuals’ personality. During this period, individuals develop the lifestyle and behavioural patterns that will last through their lifetime. For this reason, routines such as the practice of physical activity and the abandonment of sedentary behaviours should be introduced at this point in a person’s life [3]. However, abandonment of or a decrease in physical exercise is one of the problems that significantly affects adolescents, especially in developed countries [4,5]. In this regard, data on childhood obesity and the abandonment of sports has increased progressively and has become a matter of alarming social concern [6,7].

It is quite evident, then, that regular physical exercise ensures benefits for the physical, social, and mental health of all individuals. That is, it is a powerful tool for general well-being [8,9]. It is therefore worth noting the inevitable impact that motivation or barriers have on adherence to physical activity programmes. Thus, Martínez-Baena et al. [10] consider that by having a greater knowledge of different motivational profiles and barriers for the population with regard to exercise, we can put into practice more effective strategies to achieve more active participation in these type of activities.

In this work, we will consider physical exercise as a physical activity performed in a certain way with a specific objective. That is, movement is considered to be previously planned and organised in order to improve or maintain one or more aspects that determine an individual’s physical condition [11,12,13]. It is assumed that regular exercise is one of the main instruments available to the population to develop and maintain their health. Consequently; motivation, considered as the process that stimulates and directs behaviour towards the objective or goal of an activity that it instigates and maintains [13]; and barriers, understood as obstacles or impediments that lead to not carrying out an activity; have a fundamental impact on initiating and consolidating physical exercise habits [14].

A large number of works in recent years have revealed a multitude of motivational factors that intervene, either directly or indirectly, in people’s interest and participation in physical exercise. Regarding the variables that generate motivation, Rodríguez-Romo et al., [12] point to fun and occupation of free time, maintaining physical fitness and appearance, health and an enjoyment of sports, in that order, as the main reasons why people do physical exercise in their free time. They emphasise motivational differences with respect to gender and affirm that men seem to do exercise, above all, for fun and to occupy their free time, while women do it mostly to keep fit and for their figure [15]. In addition, girls stop doing exercise sooner. According to the review by Escribano et al. [16], the most common motivations among high school students, for both boys and girls, were aspects related to health and strengthening relationships. However, for boys the main reasons were fun, followed by exercising and being with friends, while fun and exercise and enjoyment of sport are the aspects that most motivate girls.

There are also significant differences between gender when it comes to knowing which people have most influenced their decision making. Fathers and friends have a positive influence on boys, while mothers and physical education teachers influence girls the most [17]. In general, positive parenting styles and tangible supports in adolescents play an important role in motivating adolescents to perform physical exercise [18]. 

In this regard, we consider it important to highlight the study by Samperio et al. [19]. The authors analysed the perceived motivational climate that involves the task of the parents and this positively predicted incremental beliefs, which in turn positively predicted the need for competition, and finally, more self-determined motivation negatively predicted barriers to physical exercise. This partially agrees with the study by Escribano et al. [16], who affirm that if both parents are physically active, their children will also be active during their free time, and vice versa. In the school environment, Villarino et al. [20] emphasise that the more positive the attitude toward physical education, the greater the practice of physical activity. The study by Baena-Extremera et al. [21] indicates that focusing more on the teaching process than on the results, encouraging personal improvement, and taking into account the effort made by students will increase the probability of achieving a more self-determined motivation in their students, with all the benefits that this entails.

Similarly, differences can be highlighted depending on the motivations. In the study by Jodkowska et al. [22], individuals who exercise show greater intrinsic motivation than sedentary subjects, which is related to adherence to exercise. They affirm that those who exercise obtained higher scores than the sedentary ones in terms of enjoyment, stress management, and improvement of their physical condition. On the other hand, sedentary individuals showed higher scores only in motivational factors related to health pressures. In the case of barriers, factors such as attitude towards sports, perceived competition, the influence of friends and colleagues, and the provision of physical education and sports in schools are aspects that can cause young people to abandon sports [23]. The barriers perceived for physical exercise among adolescents have a strong negative impact on the recommended level of physical exercise. For girls, lack of skills is the strongest predictor of low physical exercise, while for boys it is lack of time [22]. 

Martínez-Baena et al. [24] conclude that schoolchildren with high motivation towards physical exercise perceived lower levels of barriers for body image/physical–social anxiety, fatigue/laziness, and obligations/lack of time than students with moderate motivation towards physical exercise. Likewise, in the study by Samperio et al. [19] it was found that more self-determined motivation negatively predicted barriers to the practice of physical exercise.

Other studies, however, indicate socioeconomic status, marital status, educational level, and self-perceived health as factors that influence the motivation to perform physical exercise [25]. In fact, the practice of physical exercise is a complex and multidimensional behaviour determined by numerous biological, psychological, sociocultural, and environmental factors [26]. 

In this context, there is evidence that physical exercise performed during adolescence is presented at levels that do not generate health benefits, thus maintaining this situation over time [27]. Since we are concerned about this, the objective of this study was to determine adolescents’ attitudes regarding the practice of physical exercise and the relationship between difficulties and motivations to achieve physical exercise habits.

## 2. Methods

The data collected in official statistics show that an estimated total of 89,184 students were enrolled in Compulsory Secondary Education and Baccalaureate studies in the Autonomous Community of Galicia during the 2015–2016 academic year [28]. According to the STATS program, the appropriate sample size (95% confidence, 5% error, and *p* = 0.5) would be 387 individuals. Finally, the sample was of 852 students (larger than the necessary sample size obtained by the STATS program) who study secondary education and Baccalaureate, 48.9% of whom were female and 51.1% of whom were male (age = 14.86 ± 1.67 years). The distribution of the students by cycle was similar: 274 students from the first cycle of secondary education (32.2%), 328 students from the second cycle of secondary education (38.5%), and 250 Baccalaureate students (29.3%).

### 2.1. Evaluation Instruments

The instrument used to evaluate motivations was the Self-Report of Reasons for the Practice of Physical Exercise (AMPEF) questionnaire, adapted to Spanish by Capdevila et al. [29]. It consists of 48 items grouped into eight factors: Prevention and positive health (P/PH), competition, social recognition and challenge (C/SR/C), weight and body image (W/BI), affiliation, fun and well-being (A/F/W), stress control (SC), muscle strength and endurance (MS/E), health emergencies (HE), and agility and flexibility (A/F). The answer format is a Likert-type scale from 0 (not at all true) to 10 (totally true). The questionnaire shows good reliability (global scale α = 0.96, P/PH α = 0.96, C/SR/C α = 0.96, W/BI α = 0.96, A/F/W α = 0.96, SC α = 0.96, MS/E α = 0.96, HE α = 0.96, A/F α = 0.96).

To analyse the perceived barriers or difficulties in relation to physical exercise, the Self-Report of Barriers to the Practice of Physical Exercise (ABPEF) questionnaire was used, adapted by Ninerola et al. [30]. This version consists of 17 items that are answered on a Likert scale of 0 (an unlikely reason preventing me from doing physical exercise in the coming weeks) to 10 points (a very likely reason preventing me from doing physical exercise). The original study reports four different subscales: Body image/physical and social anxiety (BI/PSA), fatigue/laziness (F/L); obligations/lack of time (O/LT), and environment/facilities (E/F). This questionnaire shows good reliability (global scale α = 0.96, BI/PSA α = 0.96, F/L α = 0.96, O/LT α = 0.96, E/F α = 0.96).

### 2.2. Procedures

Once the sample and the questionnaires that were to be used were known, we contacted the management team and counsellors of the schools with the aim of explaining the purpose and scope of the research and proposing their voluntary participation. Afterwards, when the management had given their consent, an informative meeting was held to explain the study to the entire educational community. The tutors participated voluntarily and unpaid. At the same time, the parents were informed about the research and their informed consent was obtained for their children to participate in the study. The same explanatory instructions were given by the same interviewer in all the classrooms in order to avoid a bias factor. This phase of the study lasted approximately two months, from administration to collection of all the questionnaires. It is necessary to point out the receptivity of all the teachers, students, and parents whose collaboration was requested, as well as the high participation of students in our research. The ethical research protocols were complied with, especially with respect to the principles of confidentiality of the information provided and the anonymity of the subjects studied.

### 2.3. Data Analysis

This descriptive, cross-sectional, correlational study seeks to discover and explore, at a specific time, the level of motivation and barriers to the practice of physical activity faced by students who were in Compulsory Secondary Education and Baccalaureate, and in turn, to describe the relationship between them. On the one hand, a descriptive analysis (means and standard deviations) and correlational analysis (Pearson correlations) were carried out to discover the linear association between the variables. In addition, a t-test for independent samples was performed to obtain the results regarding the main effects of the interaction with gender and the effect size was calculated (Cohen’s d): Values between 0.2 and 0.3 indicate a small effect, around 0.5 a medium effect, and greater than 0.8 a high effect. For the coding and analysis of the data, the statistical program SPSS version 21 (IBM, Chicago, IL, USA) was used. 

## 3. Results

The students in the sample reported (Table 1) that the motivational factor with the highest average value was competition, social recognition, and challenge (M = 44.46 points), followed by affiliation, fun, and well-being (M = 34.71 points), and prevention or positive health (M = 33.71 points). On the other hand, the motivational factors with the lowest average value are agility and flexibility (M = 11.98), stress control (M = 11.07 points), and health emergencies (M = 10.29 points).

A positive and significant correlation is observed between all the motivational factors (see Table 1). The correlation shows a moderate and considerable relationship (r between 0.410 and 0.743) between all the variables, except between affiliation/fun/well-being and weight/body image, which is low with an effective relationship (r = 0.307). Likewise, the students in the sample give fatigue/laziness as the barrier to physical exercise with the highest average value (M = 16.07 points), followed by obligations/lack of time (M = 11.31 points), body image/physical–social anxiety (M = 10.71 points) and finally, the environment/facilities (M = 3.69) (Table 2 points).

Here, a positive and significant correlation is observed between all the factors that make up the barriers to the practice of physical exercise (see Table 2). In addition, there is a moderate correlation and a considerable relationship between fatigue/laziness and body image/physical–social anxiety (r = 0.551), although it is higher than the other factors (r between 0.225 and 0.393). 

In the correlational analysis between motivational factors and barriers to physical exercise, the relationship is very low or absent (Table 3). Thus, the correlation is positive and low, but effective, between the factors of weight/body image and body image/physical-social anxiety (r = 0.319), and negative between affiliation/fun/well-being and fatigue/laziness (r = −0.318). There is also a positive or negative but very small correlation with an almost negligible relationship between the other factors (positive: r between 0.071 and 0.159; negative: r between −0.078 and −0.180), except between prevention/positive health and body image/physical-social anxiety, obligations/lack of time, and environment/facilities; affiliation/fun/well-being and environment/facilities; stress control and fatigue/laziness; muscle strength/endurance and body image/physical-social anxiety and obligations/lack of time; health emergencies and fatigue/laziness and obligations/lack of time; and agility/flexibility and body image/physical-social anxiety, obligations/lack of time, and environment/facilities, for which there is no correlation.

The inferential analysis carried out according to gender (Table 4) presents significant differences in motivation towards the practice of physical exercise in the following factors: Competition/Social Recognition/Challenge (t (1851) = 38.60; *p* < 0.001), which is better in the male gender (medium effects size *d* = 0.426); weight/body image (t (1851) = 3.96; *p* < 0.05), which is better in the female gender (small effect size *d* = 0.116); affiliation/fun/well-being (t (1851) = 27.99; *p* < 0.001), which is better in the male gender (medium effect size *d* = 0.362); muscle strength/endurance (t (1851) = 28.47; *p* < 0.001), which is better in the male gender (medium effect size *d* = 0.365); health emergencies (t (1851) = 4.92; *p* < 0.05), which is better in the male gender (small effect size *d* = 0.151); and agility/flexibility (t (1851) = 3.95; *p* < 0.05), which is better in the female gender (small effect size *d* = 0.271).

In the same way, there are significant differences according to gender in the barriers to physical exercise (Table 4) in the following factors: Body image/physical–social anxiety (t (1851) = 20.98; *p* < 0.001), which is better in the female gender (medium effect size *d* = 0.315); fatigue/laziness (t (1851) = 23.49; *p* < 0.001), which is better in the female gender (medium effect size *d* = 0.333); and obligations/lack of time (t (1851) = 10.11; *p* < 0.05), which is better in the female gender (small effect size *d* = 0.219).

## 4. Discussion

The benefits provided by physical exercise have always been an enormously attractive topic in the field of health and quality of life, especially in the adolescent period, in which there is evidence of a loss of adherence to physical activity and sport [10,31]. For this reason, the main objective of this study was to discover the motivations or barriers that lead to greater practice or abandonment of physical exercise among adolescents, which would help to consolidate physical exercise habits that last into adulthood [32].

The results obtained show that adolescents give competition, social recognition, challenge, affiliation, fun or well-being as the main reasons for doing physical exercise, and fatigue, laziness, obligations or lack of time as the main barriers that prevent them from doing exercise. These results coincide with those reported in previous studies [17,24]. Thus, at a motivational level it can be concluded that the determining factor in doing regular physical exercise is being able to compete, have some social recognition or meet challenges [33]. In addition, the results confirm a significant relationship between the factors that constitute the motivational construct (r between 0.307 and 0.743, *p* < 0.001).

In relation to gender, boys are more motivated than girls when it comes to doing physical exercise [34], except in relation to weight and body image and agility or flexibility, which is superior in the female gender. However, it is the girls who show the greatest barriers to doing physical exercise. Our results do not match those found by Kantanista et al., [35] which revealed that body image was a statistically significant positive predictor of vigorous physical activity for adolescents, regardless of body mass index. In addition, body image was a stronger predictor in boys than in girls. Specifically, girls do not perform adequate physical activity and spend more time in sedentary activities [36]. However, both genders agree on prevention/positive health, weight and body image, stress control, and agility/flexibility as motivating factors. In this case, our study only partially agrees with [36] in which women were more motivated by weight control, appearance, agility, positive health, and stress management, and men were motivated by performance and the ego-oriented factors, such as challenge, strength and resistance, competence, affiliation, and social recognition [37]. Similarly, in the area of barriers that affect the abandonment of physical exercise, fatigue or laziness is noted as the main factor [38], giving less consideration to the environment or facilities. Other studies cite lack of motivation, sedentary behaviour given the assiduity of adolescents to the use of electronic resources, depressed mood, physical health, and school workload as barriers [39]. In addition, a significant, positive and moderate relationship was found (r between 0.225 and 0.551, *p* < 0.001) between the four factors (BI/PSA, F/L, O/LT, E/F).

On the other hand, with regard to the relationship between the motivational factors (P/PH, C/SR/C, W/BI, A/F/W, SC, MS/E, HE, A/F) and barriers to physical exercise (BI/PSA, F/L, O/LT, E/F), the results reveal very low correlations or no link between these factors. These results have been observed in other studies that show positive but very low correlations between motivational factors and barriers to physical exercise [29,30]. It can be seen that the magnitude of the correlations obtained between the motivational factors and barriers is insignificant or null. Therefore, it is considered that although they are both influenced, they each constitute different constructs.

## 5. Conclusions

In light of these results, it would be important to verify the intrinsic or extrinsic reasons for the motivational factors and barriers that encourage or prevent the practice of physical activity. For this purpose, the complementarity of this type of (qualitative) cross-sectional studies with a longitudinal design would provide valuable information for interpreting these results.

## Figures and Tables

**Table 1:** Means, standard deviations, and correlations of motivational factors towards the practice of physical exercise.

Factor	M	SD	P/PH	C/SR/C	W/BI	A/A/W	SC	S/ME	HE	A/F
P/PH	33.71	10.56	1.000							
C/SR/C	44.46	16.80	0.573 **	1.000						
W/BI	26.59	12.35	0.507 **	0.443 **	1.000					
A/A/W	34.71	12.94	0.678 **	0.743 **	0.307 **	1.000				
SC	11.07	5.57	0.625 **	0.520 **	0.435 **	0.509 **	1.000			
S/ME	16.90	7.17	0.681 **	0.703 **	0.465 **	0.663 **	0.500 **	1.000		
HE	10.29	6.39	0.494 **	0.509 **	0.410 **	0.412 **	0.466 **	0.417 **	1.000	
A/F	11.98	5.30	0.596 **	0.584 **	0.488 **	0.529 **	0.429 **	0.622 **	0.443 **	1.000

Note: P/PH (prevention/positive health); C/SR/D (competition/social recognition/challenge); W/BI (weight/body image); A/A/W (affiliation/amusement/well-being); SC (stress control); S/ME (strength/muscle endurance); HE (health emergencies); A/F (agility/flexibility) ** The correlation is significant at a level of 0.01.

**Table 2:** Means, standard deviations, and correlation coefficients of the factors that make up the barriers to the practice of physical exercise.

Factor	M	SD	BI/PSA	F/L	O/LT	E/I
BI/PSA	10.71	9.55	1.000			
F/L	16.07	9.47	0.551 **	1.000		
O/LT	11.31	6.61	0.225 **	0.371 **	1.000	
E/I	3.69	2.71	0.393 **	0.373 **	0.290 **	1.000

Note. BI/PSA (body image/physical–social Anxiety); F/L (fatigue/laziness); O/LT (obligations/lack of time); E/I (environment/Facilities) ** Correlation is significant at a level of 0.01.

**Table 3:** Correlation coefficients between the factors that make up the motivation and the barriers to the practice of physical exercise.

Motivation/Barriers	BI/PSA	F/L	O/LT	E/F
P/SP	−0.020	−0.180 **	0.050	0.016
C/RS/D	−0.003	−0.165 **	0.083 *	0.131 **
W/BI	0.319 **	0.130 **	0.114 **	0.106 **
A/F/W	−0.142 **	−0.318 **	−0.107 *	−0.021
SC	0.084 *	−0.052	0.147 **	0.078 *
S/MR	−0.023	−0.159 **	−0.025	0.071 *
HE	0.159 **	0.035	0.017	0.090 **
A/F	0.010	−0.078 *	0.012	0.030

Note. P/SP (prevention/positive health); C/SR/C (competition/social recognition/challenge); W/BI (weight/body image); A/F/W (affiliation/fun/wellness); SC (stress control); S/MR (strength/muscle resistance); HE (health emergencies); A/F (agility/flexibility); BI/PSA (body image/physical–social anxiety); F/L (fatigue/laziness); O/LT (obligations/lack of time); E/F (environment/facilities). ** Correlation is significant at a level of 0.01. * Correlation is significant at a level of 0.05.

**Table 4:** Standard deviations, independent t test, effect size (*d*) of the motivations, and barriers according to gender.

**Motivations**	**Female** **(n = 435)**	**Male** **(n = 417)**	**t** **(1851)**	***p***	***d***	**Prevalence**
	**M (DT)**	**M (DT)**				
P/SP	34.17 (10.52)	33.22 (10.59)	1.75	0.186	0.09	Ns
C/SR/C	41.03 (16.65)	48.04 (16.23)	38.6	0	0.426	>Male
W/BI	27.29 (12.51)	25.86 (12.14)	3.96	0.041	0.116	>Female
A/F/W	32.45 (13.02)	37.07 (12.44)	27.99	0	0.362	>Male
SC	10.91 (5.52)	11.23 (5.62)	1.71	0.399	0.057	ns
S/MR	15.64 (7.04)	18.22 (7.08)	28.47	0	0.365	>Male
HE	9.81 (5.54)	10.78 (7.14)	4.92	0.027	0.151	>Male
A/F	12.59 (5.39)	11.15 (5.20)	3.95	0.04	0.271	>Female
**Barriers**	**Female** **(n = 435)**	**Male** **(n = 417)**	**t** **(1851)**	***p***	***d***	**Prevalence**
	**M (DT)**	**M (DT)**				
BI/FSA	13.16 (10.61)	10.19 (8.03)	20.98	0	0.315	>Female
F/L	17.59 (9.98)	14.48 (8.65)	23.49	0	0.333	>Female
O/LT	12.01 (6.57)	10.57 (6.58)	10.11	0.002	0.219	>Female
E/F	3.63 (2.58)	3.76 (2.82)	1.46	0.46	0.048	ns

Note: ns = not significant. P/SP (prevention/positive health); C/SR/C (competition/social recognition/challenge); W/BI (weight/body image); A/F/W (affiliation/fun/wellness); SC (stress control); S/MR (strength/muscle resistance); HE (health emergencies); A/F (agility/flexibility); BI/PSA (body image/physical–social anxiety); F/L (fatigue/laziness); O/LT (obligations/lack of time); E/F (environment /facilities).
